# Assessing a GPS-Based 6-Minute Walk Test for People With Persistent Pain: Validation Study

**DOI:** 10.2196/46820

**Published:** 2024-03-18

**Authors:** Joshua Simmich, Nicole Emma Andrews, Andrew Claus, Megan Murdoch, Trevor Glen Russell

**Affiliations:** 1 RECOVER Injury Research Centre Faculty of Health and Behavioural Sciences The University of Queensland Brisbane Australia; 2 STARS Education and Research Alliance Surgical Treatment and Rehabilitation Service The University of Queensland and Metro North Health Brisbane Australia; 3 The Tess Cramond Pain and Research Centre Royal Brisbane and Women's Hospital Metro North Hospital and Health Service Brisbane Australia; 4 Occupational Therapy Department Royal Brisbane and Women's Hospital Metro North Hospital and Health Service Brisbane Australia; 5 School of Health and Rehabilitation Sciences The University of Queensland Brisbane Australia; 6 Physiotherapy Department Royal Brisbane and Women's Hospital Metro North Hospital and Health Service Brisbane Australia

**Keywords:** GPS, mobile apps, exercise test, pain, chronic pain, mobile phone

## Abstract

**Background:**

The 6-minute walk test (6MWT) is a commonly used method to assess the exercise capacity of people with many health conditions, including persistent pain. However, it is conventionally performed with in-person supervision in a hospital or clinic, therefore requiring staff resources. It may also be difficult when in-person supervision is unavailable, such as during the COVID-19 pandemic, or when the person is geographically remote. A potential solution to these issues could be to use GPS to measure walking distance.

**Objective:**

The primary aim of this study was to assess the validity of a GPS-based smartphone app to measure walking distance as an alternative to the conventional 6MWT in a population with persistent pain. The secondary aim of this study was to estimate the difference between the pain evoked by the 2 test methods.

**Methods:**

People with persistent pain (N=36) were recruited to complete a conventional 6MWT on a 30-m shuttle track and a 6MWT assessed by a smartphone app using GPS, performed on outdoor walking circuits. Tests were performed in random order, separated by a 15-minute rest. The 95% limits of agreement were calculated using the Bland-Altman method, with a specified maximum allowable difference of 100 m. Pain was assessed using an 11-point numerical rating scale before and after each walk test.

**Results:**

The mean 6-minute walk distance measured by the GPS-based smartphone app was 13.2 (SD 46; 95% CI −2.7 to 29.1) m higher than that assessed in the conventional manner. The 95% limits of agreement were 103.9 (95% CI 87.4-134.1) m and −77.6 (95% CI −107.7 to −61) m, which exceeded the maximum allowable difference. Pain increased in the conventional walk test by 1.1 (SD 1.0) points, whereas pain increased in the app test by 0.8 (SD 1.4) points.

**Conclusions:**

In individuals with persistent pain, the 2 methods of assessing the 6MWT may not be interchangeable due to limited validity. Potential reasons for the differences between the 2 methods might be attributed to the variation in track layout (shuttle track vs continuous circuit); poor GPS accuracy; deviations from the 30-m shuttle track; human variability in walking speed; and the potential impact of a first test on the second test due to fatigue, pain provocation, or a learning effect. Future research is needed to improve the accuracy of the GPS-based approach. Despite its limitations, the GPS-based 6MWT may still have value as a tool for remote monitoring that could allow individuals with persistent pain to self-administer frequent assessments of their functional capacity in their home environment.

## Introduction

### Background

Persistent pain affects 20% of the Australian population, has fueled the opioid epidemic, and cost the Australian government Aus $73.2 billion (US $54.7 billion) in 2018, the latest year for which figures are available [1,2]. People with persistent pain report that their pain negatively affects their ability to exercise and their physical functioning [3].

The 6-minute walk test (6MWT) is a standard method for measuring submaximal exercise capacity, which measures the distance a person can walk over 6 minutes (6-minute walk distance; 6MWD). Conventionally, the 6MWT involves the participant walking multiple laps of a straight path that is 30 m in length [4]. Track length can influence the results of the 6MWT [5-8], likely due to a difference in the number of 180-degree turns, each necessitating acceleration and deceleration. Standard instructions are given and standard encouragement prompts are provided at preset intervals, as it is known that changes in instructions or encouragement can affect the performance during this test [9,10].

The 6MWT has been used in a range of conditions including chronic heart failure [11], peripheral artery disease [12], and chronic obstructive pulmonary disease [13]. It has also been used for older adults more generally [14]. Within the context of pain conditions, the 6MWT has been used as an outcome measure in both observational studies [15] and clinical trials [16,17]. The average 6MWD for people with persistent pain upon entry into pain programs has been reported to be 389 (SD 94) m [18] or 427 (SD 127) m [19]. In comparison, the reference values of the 6MWD for healthy men and women aged between 50 and 60 years are approximately 578 m and 534 m, respectively [20].

The minimum clinically important difference (MCID) for the 6MWD has been estimated for various conditions: 25 to 80 m in people with chronic obstructive pulmonary disease [21-23]; 22 to 42 m in people with lung cancer [24]; 33 m in people with pulmonary arterial hypertension [25]; 36 m in people with chronic heart failure [26]; 25 m in people with coronary artery disease [27]; and 18 m in older adults at risk of falls [28]. However, estimates in the population with persistent pain are less well defined, with different studies providing a range of estimates from a distance between 15 m and 30 m used in a clinical trial [16] to a distance between 156 m and 167 m calculated in fibromyalgia (a persistent pain disorder) [29]. Benaim et al [19] used an anchor-based approach to estimate MCID values of 60 m for chronic musculoskeletal pain of the spine and 75 m for chronic musculoskeletal pain of the lower limb. On the other hand, a recent study was unable to calculate an MCID in people with persistent pain undergoing a pain rehabilitation program due to a lack of correlation between the 6MWD and patient-reported outcome measures [18]. Therefore, further studies are needed to determine a more precise and consistent estimate of MCID values for the 6MWD in the population with persistent pain.

The conventional 6MWT is typically performed in a clinic or hospital. However, this is a significant limitation if patients cannot attend the facility due to distance or lack of access to transport. In addition, it can be difficult for some facilities to locate a straight section of corridor that is at least 30 m in length and largely free of obstacles and interruptions from other users of the corridor. The 6MWT can be performed outdoors, as an outdoor 6MWT appears to be comparable to an indoor 6MWT when the standard 30-m track is used [30], although outdoor testing can be limited by weather at the time of the test. The 6MWT can also be conducted at a participant’s residence, but this requires the clinician to travel to the patient, which increases the requirement for staff time and resources. In addition, locating a suitable 30-m track inside a house or in a backyard can be difficult. Holland et al [31] attempted to perform the 6MWT using the longest length of track that was practicable in the home environment but achieved an average track length of just 13 (SD 7) m for indoor tests and 20 (SD 10) m for outdoor tests. As such, the 6MWD estimates obtained from 6MWTs conducted inside or outside a home underestimated the 6MWD by an average of 30 (SD 69) m compared to the standard in-clinic tests using a 30-m track [31]. The variability in track length also contributed to wide limits of agreement (LoAs) between a conventional 6MWT conducted at a hospital and a 6MWT conducted at home (95% LoA 167 m-102 m). Finally, any method of performing the 6MWT that requires a clinician to assess the participant in person may be precluded if in-person contact needs to be limited, such as occurred worldwide during the COVID-19 pandemic. This has highlighted the need for alternative methods of performing the 6MWT.

Performing the 6MWT remotely would eliminate the problems associated with travel or in-person contact. Several studies have reported the use of accelerometers in smartphones or consumer-level wearable activity monitors to perform 6MWTs by detecting the number of steps or number of turns [32-38]. For instance, in a study conducted among healthy adults, the mean difference between accelerometer-based measurement and simultaneous clinician observation of a conventional 6MWT was 0, although the SD was 42 m [32]. However, this has not been replicated in clinical populations, and the app is not publicly available. Other accelerometer studies required their participants to use a holster or harness to affix their smartphone to a specific location on their body [38] or to wear a wrist-worn activity tracking device [33], both of which are potential barriers to use or uptake in a home environment. Furthermore, the 6MWT assessed via an app would ideally be undertaken on a 30-m-long track to best replicate the clinical setting, but it is unlikely that people with persistent pain would have access to such a length of track or the equipment to precisely measure its length in the home environment.

A potential solution to these issues could be to use GPS to measure the walking distance [39]. Modern smartphones include GPS receivers, and GPS-based walk tests can be conducted during an outdoor walk over level terrain (given this walk is free from the obstructions of tall buildings and does not involve many sharp turns). Although changing the 6MWT from the conventional shuttle to a continuous walk introduces some bias due to the removal of acceleration and deceleration when turning around, this might be less than the bias that would occur from using very short track lengths. For instance, in a population with respiratory problems, when compared to a 6MWT performed on a 30-m shuttle track, a 6MWT performed on a continuous circular track of 40-m circumference overestimates the 6MWD by 3% [40], but decreasing a shuttle track to 10 m underestimates the 6MWD by approximately 9% to 10% [8]. Furthermore, it may be possible to use an algorithm to compensate for the overestimation associated with continuous walking if it is consistent, but this is unlikely for the variable error that is associated with using tracks of varying and imprecisely measured lengths in a home environment.

Salvi et al [36] recently developed an app, called Timed Walk, which could use either an indoor algorithm using smartphone sensors or an outdoor algorithm using GPS. To the best of our knowledge, this is the only purpose-built app for conducting a 6MWT that is available on both Android and Apple iOS smartphones and the only app that uses GPS. This app has been tested in a population with pulmonary arterial hypertension in 2 studies that estimated the bias (the average difference between the app’s measurement and a reference) and the variability of the differences (given as SD of the differences). In the first study, Salvi et al [36] used a distance wheel as the reference measurement and found a larger bias but smaller variability when using the indoor algorithm (mean −2.0, SD 7.8 m) than when using the outdoor algorithm (mean −0.80, SD 18.6 m). However, in their later pilot trial, the indoor algorithm differed by 14.6 (SD 75) m when compared to the simultaneous performance of a conventional 6MWT in a clinic. This difference and SD is greater than that of the outdoor algorithm (mean 2.5, SD 47 m) compared to a clinic-based test performed within a 7-day period (before or after) [41]. The authors believe that much of this variation can be attributed to the app being used incorrectly, such as using the outdoor test on a tightly curved path. However, these studies compared the outdoor GPS mode of the app to a distance wheel [36] or to a conventional 6MWT performed within a 7-day period (before or after) [41]. Thus, interday variation in submaximal exercise capacity could also have contributed to the variation between these measurements. A comparison between the GPS-based test function of this app and a conventional 6MWT performed on the same day has not been attempted. In addition, this app has not been validated in the population with persistent pain.

### Aims and Hypothesis

The primary aim of this study was to evaluate the concurrent validity of the GPS-based 6MWT using the Timed Walk smartphone app in an outdoor setting and conducted on the same day as the conventional 6MWT using a 30-m straight path. The secondary aim of this study was to estimate the difference between the pain evoked by the 2 test methods.

It was hypothesized that the agreement between the results of the 2 methods will be within the nominated maximum allowable difference and that the 2 methods will demonstrate an excellent level of correlation.

## Methods

This was an observational study comparing 2 methods of estimating the 6MWD: a conventional 6MWT with a clinician and a 6MWT using a smartphone app with GPS.

### Participants

The sample comprised a combination of participants recruited from 2 sources, to ensure a broad sample of people with persistent pain. The first recruitment method involved outpatients from a persistent pain clinic located in a large, tertiary public hospital in Australia. Potential participants were invited to participate in the study by their treating clinician. The second method of recruitment involved advertising the study via social media (Facebook and Twitter).

Participants were screened via an in-person or telephone conversation with a member of the research team. Individuals were eligible for inclusion if they were aged >18 years, had persistent pain (>9 months in duration), claimed that they were able to walk at least 100 m on flat ground, and owned a compatible smartphone on which they were willing to install the Timed Walk app. Individuals were excluded from participating if they had comorbidities impacting their ability to walk on flat ground (and did not have medical clearance to participate), were unable to speak English, or did not possess a compatible smartphone.

### Data Collection

Participants performed 2 6MWTs: one in the conventional manner with a clinician and another with the Timed Walk app (version 0.2.0 or 0.3.0, depending on the date of the test).

The app test was conducted outdoors to use the GPS signal. Participants were instructed to walk either in a circuit around the paths of a local park or around the perimeter of a hockey playing field for the app test. Care was taken to select a mostly level path and to avoid sharp turns. As walking alongside a participant can influence the results of a 6MWT [42], a researcher walked at a comfortable distance behind each participant to provide supervision. The Timed Walk app automatically announced the standard encouragement prompts at appropriate times.

The 6MWT assessed in the conventional manner was completed in accordance with existing guidelines [4], using a straight track of 30 m in length, with the ends of the track marked with cones and the starting line marked with a clearly visible line on the ground. A researcher (JS) conducted all the tests and provided the standard encouragement prompts.

For practical reasons and to minimize differences between the testing methods, the conventional 6MWT was also conducted outdoors at the same location as the GPS test. Outdoor testing has been shown to be no different to indoor testing in individuals with pulmonary disease [30]. Outdoor testing was only performed when weather conditions were mild (temperature of 10 °C-30 °C, no rain, and wind speed <20 km/h).

To account for potential fatigue or learning effects, the sequence in which the participants performed each form of the 6MWT was randomized on the day of testing. Urn design randomization was used [43]. Participants were randomized using a simulated urn initially containing 2 balls, with 1 ball representing each test method. After drawing a ball to allocate each participant to their starting test method, the ball was returned to the urn and an additional ball added for the opposite test method. To further minimize the effect of fatigue, participants were rested in a shaded location for 15 minutes between tests. Participants were instructed to wear comfortable clothing and appropriate footwear, to be well hydrated (and bring a water bottle), and to not perform any vigorous exercise 2 hours before and after the test.

Neither the participants nor the researcher performing the conventional 6MWT were blinded to the study hypothesis or to the methods of assessment being received.

### Outcome Measures

The primary outcome of this study was the 95% LoAs between conventionally measured and GPS-measured 6MWD.

Secondary outcomes included the following: (1) the intraclass correlation coefficient (ICC) between the conventionally measured and GPS-based 6MWD and (2) the difference between the change in pain for the 2 types of 6MWTs. Pain intensity data were collected at four time points: (1) immediately before the first 6MWT, (2) immediately after the first 6MWT, (3) immediately before the second 6MWT, and (4) immediately after the second 6MWT. Pain was assessed on an 11-point numerical rating scale (NRS), verbally delivered to the participant at the abovementioned time points. This scale ranged from 0 (indicating no pain at all) to 10 (indicating pain as bad as it could be or worst pain).

Additional demographic data collected included participant age, gender, employment status, primary diagnosis (including pain source or body region), and years since diagnosis of the persistent pain condition. These data were collected using a paper-based questionnaire on the day of testing.

### Statistical Analysis

The Bland-Altman method of analysis was used to estimate the 95% LoAs between the conventionally measured and GPS-measured 6MWD. The exact parametric 95% CIs for the upper and lower limits of the 95% LoAs, considered together as pairs, were calculated using the methods proposed by Carkeet [44]. The maximum allowable difference between measures was set at 100 m, approximately in between the 60-m to 75-m MCID for people with chronic musculoskeletal pain at a single site undergoing occupational rehabilitation [19] and 156-m to 167-m MCID in people with fibromyalgia [29].

Outliers were excluded from the analysis if the difference between the 2 test methods was >3 SDs from the mean difference.

Assumptions of normality and homoscedasticity were assessed through visual inspection of quantile-quantile plots and residual plots. The ICC was calculated using a 2-way random-effects model for both absolute agreement and consistency, with values >0.8 considered to be good and those >0.9 considered to be excellent. The difference between the mean change in pain due to each test was presented with paired-sample CIs.

Several exploratory analyses that were not initially planned were conducted after the results for the main outcome measures were inspected. The exploratory analysis of the variance between recruitment groups was conducted using an *F* test. Welch 2-sample CIs were used for the exploratory comparisons between the 2 smartphone operating systems (Android and iOS) and the 2 sequences in which the tests were conducted (in-person test conducted first and app test conducted first). Paired-sample CIs were used to present the results of an exploratory comparison between pain at the start of the first test and pain at the start of the second test.

Randomization and statistical analyses were performed using R software (version 4.2.1; R Foundation for Statistical Computing). ICCs were calculated using the *irr* package (version 0.84.1) [45].

### Sample Size and Power

The sample size required for the Bland-Altman analysis was calculated using the method outlined by Shieh [46]. A difference of 2.5 m was used for this calculation, based on a study comparing an unsupervised test using the GPS-based Timed Walk app performed at home and a clinic-based test conducted within a 7-day period (before or after) [41]. However, with the assumption that the tests performed on the same day and under supervision would result in slightly less variability, sample size calculation assumed an SD of 40 m rather than the previously reported value of 47 m. The maximum allowable difference between measures was set at 100 m as previously stated. Additional assumptions include the following: 90% power, α of .05, and a null central portion of 0.95 (ie, 95% of measurements lie between the 100-m maximum allowable difference boundaries). This calculation resulted in a requirement for 34 participants, which was increased to 38 participants to account for potential dropouts.

### Ethical Considerations

All participants were provided with a participant information sheet that explained the study, and they provided their written consent to participate. Participants were compensated with a voucher worth Aus $20 (US $14-15) for their time and travel expenses. All data were deidentified. The study was approved by the Royal Brisbane and Women’s Hospital Human Research Ethics Committee (reference number HREC/2021/QRBW/75331) and ratified by the University of Queensland Human Research Ethics Committee (reference number 2021/HE001471).

## Results

### Sample Description

A total of 38 participants (n=17, 45% from the community and n=21, 55% from the outpatient pain clinic) were eligible and consented to participate. Of the 38 participants, 1 (3%) withdrew due to illness, 1 (3%) was excluded for no longer meeting the inclusion criteria, and 1 (3%) participant, whose GPS-based 6MWD was 88% more than their conventional 6MWD, was considered as an outlier and excluded from the analysis. The first participant was tested on October 27, 2021, and the final participant was tested on August 27, 2022 (reflecting issues with recruitment during the COVID-19 pandemic). A full description of the final sample of participants (N=35) is presented in Table 1.

**Table 1:** Description of individual samples and the combined sample.

Characteristics		Community (n=16)	Outpatient pain clinic (n=19)	Total (N=35)
Age (y), mean (SD)		50 (16)	49 (12)	49 (13)
**Gender, n (%)**				
	Women	14 (88)	15 (79)	29 (83)
	Men	2 (13)	4 (21)	6 (17)
**Type or location of pain, n (%)**				
	Low back pain	8 (50)	9 (47)	17 (49)
	Whole body pain or fibromyalgia	2 (13)	8 (42)	10 (29)
	Lower limb pain	4 (25)	1 (5)	5 (14)
	Neck or upper limb pain	2 (13)	1 (5)	3 (9)
Years since pain onset, mean (SD)		13 (15)	13 (11)	13 (13)
Baseline pain score (0-10 on NRS^a^), mean (SD)		2.9 (2.2)	5.1 (2.5)	4.1 (2.6)
**Smartphone operating system, n (%)**				
	Android	9 (56)	15 (79)	24 (69)
	Apple iOS	7 (44)	4 (21)	11 (31)

^a^NRS: numerical rating scale.

After randomization, of the 35 participants, 17 (49%) participants performed the GPS-based 6MWT first and the conventional 6MWT second and the remaining 18 (51%) participants performed the tests in the reverse order. The results for the 2 types of 6MWTs, according to the order of the tests, are presented in Table 2.

**Table 2:** Values for the 6-minute walk distance (6MWD) according to method of measurement and order of tests.

	First 6MWD, mean (SD)	Second 6MWD, mean (SD)	Average 6MWD, mean (SD)
GPS-based 6MWD	426.5 (132.7)	466 (148.7)	446.8 (140.5)
Conventional 6MWD	453 (134.8)	413.1 (12.5.3)	433.6 (129.9)
Average 6MWD, mean (SD)	440.1 (132.5)	440.3 (138.4)	N/A^a^

^a^N/A: not applicable.

### Concurrent Validity

**Figure 1:**
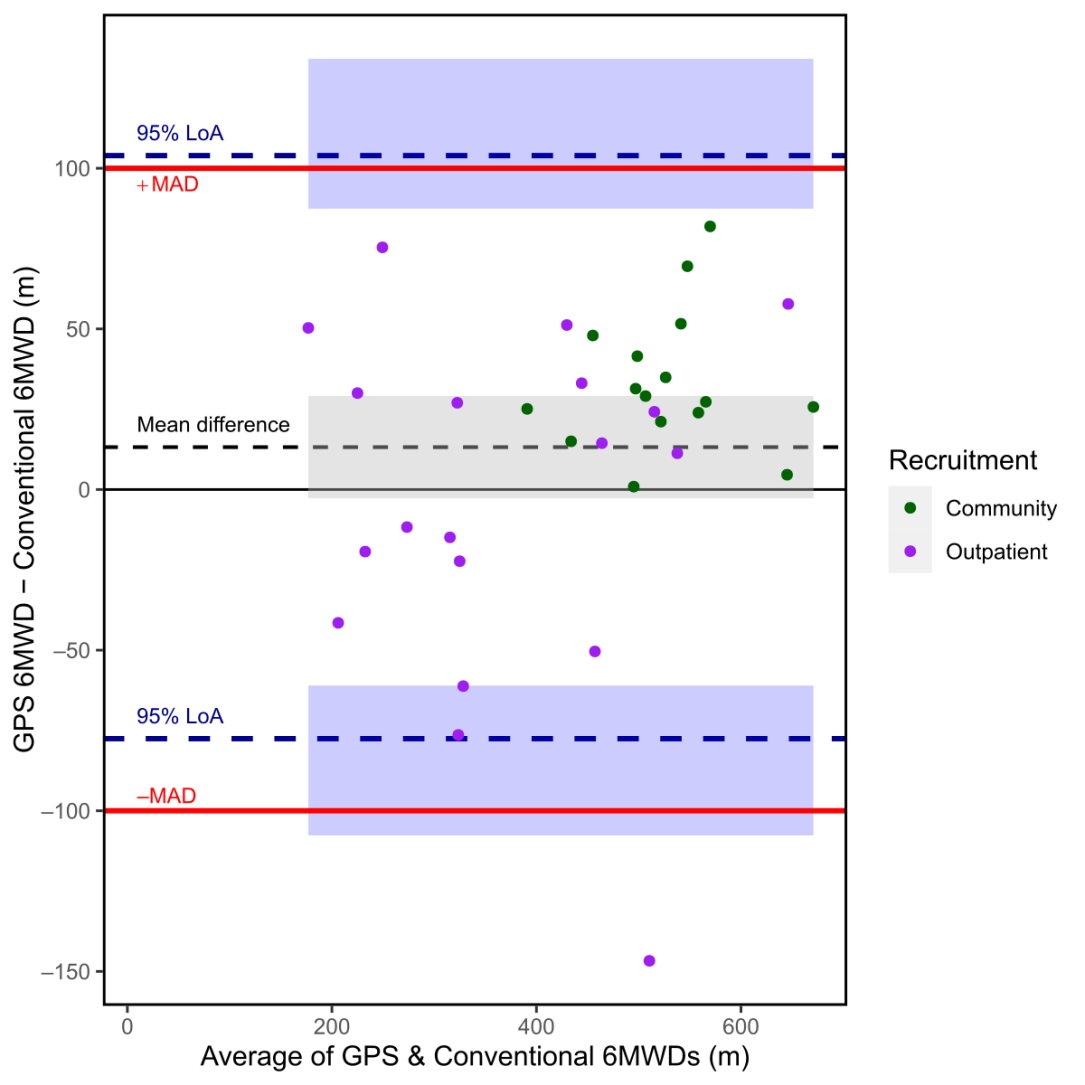
Bland-Altman plot for the GPS-based and the conventional 6-minute walk tests, with recruitment source indicated. Shaded areas represent the 95% CI for each estimated value. LoA: limits of agreement; MAD: maximum allowable difference; 6MWD: 6-minute walk distance.

The mean 6MWD measured by the GPS-based smartphone app was 13.2 m (SD 46 m; 95% CI −2.7 to 29.1) higher than the 6MWD assessed in the conventional manner. The 95% LoAs were 103.9 (95% CI 87.4-134.1) m and −77.6 (95% CI −107.7 to −61.0) m, which are not entirely within the maximum allowable difference of 100 m. A Bland-Altman plot is presented as Figure 1. As the distribution of the differences was leptokurtotic (an excess kurtosis of 2.2), an alternative, nonparametric Bland-Altman approach was applied, which yielded a median difference of 25.1 m and 95% LoAs (at the 2.5% and 97.5% quantiles) of −86.9 m and 76.4 m.

The ICC for agreement (2-way random-effects model) between the 2 6MWTs was 0.94 (95% CI 0.88-0.97) and the ICC for consistency (2-way random-effects model) was 0.94 (95% CI 0.89-0.97), both of which indicate good to excellent agreement.

The average of the two 6MWD measurements differed based on recruitment source, with those recruited from the community having a higher 6MWD (527 m, 95% CI 489-564) compared to those recruited from the outpatient clinic (368 m, 95% CI 304-431). The mean difference between the two 6MWD measurements also differed based on recruitment source, with outpatient measurements only differing by –4 m (95% CI −30 to 23) compared to a much higher difference of 33 m (95% CI 22-44) in the community sample. The apparent trend of overestimation by the GPS-based 6MWT relative to the conventional 6MWT with increased walking distance seems to be attributable to participants from the community displaying greater values than outpatients for both the average 6MWD and the difference between GPS-based and conventional 6MWD measurements (Multimedia Appendix 1). An exploratory comparison suggests that the difference between the two 6MWTs in the outpatient sample had a variance that was 6.5 (95% CI 2.3-17.4) times higher than the variance in the community sample (3009 m^2^ vs 461 m^2^).

In addition, an exploratory comparison found that the mean difference between the two 6MWT methods (GPS 6MWD minus conventional 6MWD) was 34.9 m (95% CI –5.29 to 75.2) greater when Android smartphones were used compared to that when Apple smartphones were used. The difference in 6MWDs when the GPS-based 6MWT was performed on Android smartphones was 34.2 (SD 61.6) m, whereas the difference in 6MWDs when the app test was performed on Apple smartphones was –10.8 (SD 57) m.

Another exploratory comparison suggests that the order in which the 2 tests were conducted has no clear effect on the difference between the tests. The mean difference between the two 6MWT methods (GPS 6MWD minus conventional 6MWD) when the GPS-based 6MWT was conducted first was 13.4 (SD 45.5) m, and the difference between the methods when the conventional 6MWT was performed first was 13.0 (SD 48.4) m. Therefore, when the app 6MWT was performed first, the mean difference between the tests was increased by 0.3 m (95% CI –31.9 to 32.6) versus when the conventional 6MWT was performed first.

### Change in Pain

Pain increased in the conventional walk test by 1.1 (SD 1) points, whereas pain increased in the app test by 0.8 (SD 1.4) points. The conventional walk test produced a change in pain score that was 0.26 (95% CI −0.20 to 0.73) points higher than that produced by the app test.

**Figure 2:**
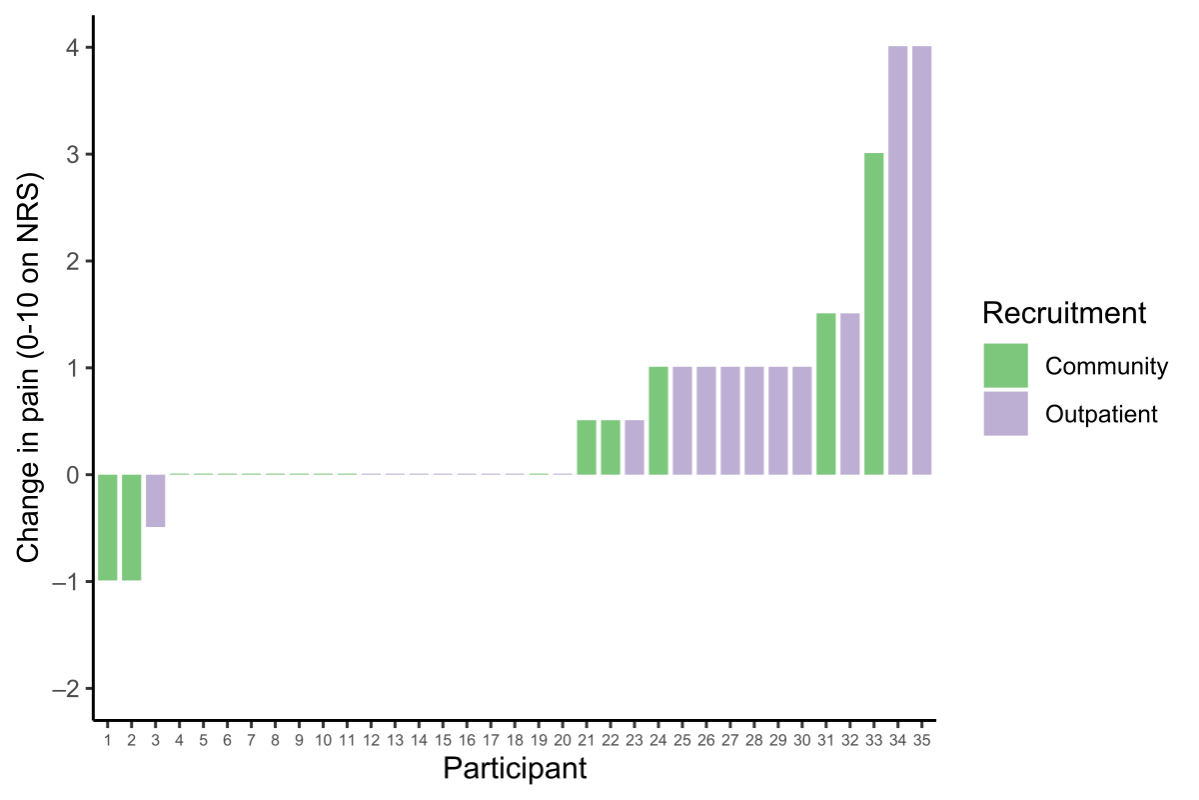
Ordered column plot of individual changes in pain from the start of the first test to the start of the second test, with the recruitment source additionally indicated. NRS: numerical rating scale.

An exploratory comparison suggests a statistically significant difference between pain at the start of the first test compared to pain at the start of the second test (t_34_=2.969; P=.005). The pain rating before the first test averaged 4.1 (SD 2.5) points, and the pain rating before the second test (after the rest period) averaged 4.7 (SD 2.6) points. On average, pain at the beginning of the second test was 0.57 (95% CI 0.18-0.96) points higher than pain at the beginning of the first test. However, the median difference in pain was 0 (range –1 to 4) points. Approximately half (17/35, 49%) of the participants reported a pain level at the start of the second test that was identical to the level reported at the start of the first test. Figure 2 illustrates the distribution of individual differences between pain ratings at the start of each test.

A final exploratory analysis was conducted to elucidate the relationship between changes in pain intensity and measured walking distances. Scatterplots were used to visualize the changes in pain intensity and measured walking distances (Multimedia Appendix 2). Apart from a single participant who experienced the largest decrease in the 6MWD (approximately 150 m) and the greatest increase in pain (4 points on NRS), we did not observe any relationship between change in pain and walking distance.

## Discussion

### Principal Findings

This study investigated the validity of a GPS-based 6MWT smartphone app (Timed Walk) compared to that of the conventional method of conducting a 6MWT in people with persistent pain. To the best of our knowledge, this is also the first study to compare the Timed Walk app to a conventional 6MWT conducted on the same day in any population.

Participants walked an average of 433.6 (SD 129.9) m during the conventional 6MWT, which is typical of people with persistent pain [15,19,47]. The mean pain score at baseline for participants of this study was 4.1 (SD 2.6), which is moderately lower than the averages of 6.0 and 6.4 noted during enrollment into a pain program in the literature [15,47]. This is likely due to our inclusion of participants from the community, who would be expected to have lower pain scores than those enrolled in a rehabilitation program. However, the baseline pain scores of the outpatients included in this study was 5.2 (SD 2.5), which is more consistent with previous estimates. Therefore, we can still be confident that the study sample was representative of people with persistent pain.

The results of the Bland-Altman analysis indicated uncertainty about the agreement between the 2 methods of measuring 6MWD. The 95% LoAs were 103.9 (95% CI 87.4-134.1) m and −77.6 (95% CI −107.7 to −61.0) m. These CIs are consistent with LoAs that are fully outside or within our a priori 100-m maximum allowable difference. Therefore, there is uncertainty about whether 95% of the differences will lie within this 100-m threshold. These LoAs are also larger than the estimated MCID in the 6MWT for chronic musculoskeletal pain, which is estimated to be 60 m for chronic musculoskeletal pain of the spine and 75 m for chronic musculoskeletal pain of the lower limb [19], but are within the much larger MCID estimate range of 156 m to 167 m for fibromyalgia [29]. Therefore, agreement between methods might not be sufficient for clinicians to use these two 6MWT methods interchangeably and still be able to reliably detect clinically important differences in the 6MWD for people with chronic pain of the limbs and spine. It is possible that the level of agreement is sufficient to detect clinically significant differences in people with fibromyalgia; however, this study was not performed specifically among people with fibromyalgia.

There are several potential reasons for differences between the two 6MWD measurements. First, the tests differed in how they were conducted, with one using a 30-m shuttle track and the other using various circuits around a public park. The GPS-based 6MWT involved fewer and more gradual turns, without the requirement to decelerate and accelerate with short turns on a shuttle track. Previous studies have demonstrated that continuous (circular or rectangular) tracks result in 6MWD estimates that are 3% to 10% higher than those obtained from a straight shuttle track [7,40,48]. Therefore, this is a likely explanation for the observed 6MWD derived from the Timed Walk app in this study being 13.2 m (95% CI −2.7 to 29.1) higher than that obtained from the conventional 6MWT—approximately a 3% increase. However, the CIs for the mean difference in the 6MWD between tests are wide enough to include both the possibility of no difference between the tests and a difference as large as approximately 30 m or approximately 7% of the total 6MWD. The 2 tests also differed in how encouragement phrases were delivered—by a researcher or through audio from the smartphone app—which could also impact walking distance. In addition, although the 30-m track was consistent for all conventional 6MWTs, the exact walking route used for the GPS-based 6MWT for each participant varied in terms of potentially influential factors such as the number of turns, radius of turns, and number of laps performed. It is possible that differences in the walking routes used could also partially explain the variability between the 2 tests.

Second, the tests differed in how they were measured, which could have introduced errors that could be observed as either systemic bias or as variability in the results. Inaccuracy of measurement in the conventional 6MWT might occur due to inaccurate measurement of the length of the track (or partial final lap), the participant not being able to stop instantly at 6 minutes, and the participant deviating from the track during walking or making wider turns around the markers. Although none of these issues are present for the GPS method of measurement, other sources of inaccuracy likely exist. In their initial 30 tests with 8 different smartphones, Salvi et al [36] compared the GPS-based algorithm of the Timed Walk app to a simultaneous measurement of distance with a trundle wheel, thereby eliminating variability between separate walking tests. Although the mean difference between those 2 measurements was minimal at 0.8 m, the SD was 18.6 m (or approximately 4.2% of the mean 6MWD of 438 m). As the Bland-Altman method calculates 95% LoAs as 1.96 SDs from the mean, approximately 36-m wide 95% LoAs (or 8.4% of the 6MWD) might be expected based on measurement error alone. This measurement error could be related to a poor-quality GPS signal (due to tree coverage or cloud cover), inaccurate GPS receivers in smartphones, and issues with the app’s algorithm. GPS accuracy is typically assessed in a static (nonmoving) condition or while driving [49,50], both of which differ significantly from a walking test. The accuracy of GPS measurements in the context of a dynamic, real-world activity such as walking is influenced by various factors, including signal processing and filtering algorithms specific to each app. Some previous studies suggest that the GPS receivers have different levels of accuracy across different models of smartphones [51]. The difference we observed between Android and Apple smartphones suggests some role for the hardware or operating system, which, as participants’ devices were effectively random, could introduce an additional source of variability.

Conducting 2 separate walking tests introduces an additional source of variability due to normal human variability in walking speed, in addition to the measurement error discussed previously. The conventional 6MWT, when performed 1 week apart, has a minimum detectable change (at 95% confidence) of 86 m in people with persistent pain [18] and from 50 m to 80 m in adults aged >60 years [52]. The minimum detectable change at 95% confidence is equivalent to the difference between the 95% LoA and the mean difference [53,54]. This suggests that the conventional method of assessing a 6MWT is only barely able to reliably detect a change of the same magnitude as our nominated 100-m maximum allowable difference. The test-retest reliability of the Timed Walk app has only been reported once in the literature. Salvi et al [41] have reported data about the test-retest reliability of the Timed Walk app in pulmonary hypertension, based on 89 pairs of outdoor tests performed 7 days apart in 10 patients. That study also reported a mean difference of 1.8 m (0.7% of the 6MWD) and an SD of 37 m (or 10.1% of the 6MWD) between pairs of GPS-based 6MWTs, giving a 95% LoA of approximately 74 m on either side of the mean. Therefore, this evidence suggests that it can be difficult to reliably detect a change of 100 m between two 6MWTs, even when both 6MWTs are conducted using the same methods (either the conventional method or the GPS-based method).

Finally, there could be some effect of the first test on the results of the second text. For instance, performance might have decreased during the second test due to the provocation of fatigue or pain. Although a 15-minute rest period is sufficient for people with cardiac disease to recover from fatigue induced by walking [55], this may not be the case for recovery from pain. We found that some (15/35, 43%) of participants reported higher pain scores at the start of their second test than at the start of their first test. This residual pain could have altered their performance during the second test, as pain can increase the energy cost of walking [56,57]. On the other hand, a learning or practice effect whereby 6MWD increases for a second 6MWT conducted on the same day has been demonstrated in several studies in healthy adults [58,59] and people with respiratory disease [60] and chronic heart failure [61]. This learning effect may be due to decreased anxiety (more willingness to walk faster if they see that it does not provoke as much pain or fatigue) or improvements in technique (optimum stride length or pacing) during subsequent tests [4]. In addition, a learning effect may still occur regardless of the rest period between tests conducted on the same day. As the order in which the 2 tests were conducted was randomized in this study, any effect of test order would manifest as variance in the difference between the 2 methods. However, when the difference between 6MWD measurements was compared between the subgroup randomized to perform the app test first versus the subgroup performing the app test second, there was no clear evidence of a difference. As the 2 walk tests investigated in this study were very dissimilar, a learning effect may have been less likely to occur. Alternatively, a learning effect could have been countered by an approximately equal and opposite effect of pain or fatigue. Interestingly, the greater variability between the 2 tests seen in the outpatient subsample, who also had higher pain scores and more widespread pain than the community subsample, may also suggest that those who are experiencing greater pain have more variable walking speeds in general. This could be related to spatiotemporal gait mechanics, which are known to be affected by pain conditions [62,63].

Validity, as assessed by the ICC for agreement (2-way random-effects model) between the 2 6MWTs, was good to excellent at 0.94 (95% CI 0.88-0.97), suggesting that approximately 12% of the variation between the 2 measurements is due to bias or error. ICC for consistency was also good to excellent at 0.94 (95% CI 0.89-0.97). The near equality of these ICC values indicates that the validity is mostly being affected by random error rather than systemic error (bias). Despite the variability introduced by the previously mentioned differences between the GPS-based and conventional 6MWTs, the ICC for agreement in this study is similar to previously reported ICC values (agreement) of 0.91 and 0.92 for the test-retest reliability (with 1 day or 7 days between tests) of the conventional 6MWT in fibromyalgia [64,65]. Similarly, it is comparable to the ICC of 0.91 reported by Salvi et al [41] for the test-retest reliability of the Timed Walk app in pulmonary hypertension, based on 89 pairs of outdoor tests performed 7 days apart in 10 patients.

### Pain

Secondarily, the study also compared the 2 methods to examine the differences in the degree of pain evoked by the test. Pain increased by approximately 1 point on NRS in both tests. Therefore, there is no indication of any additional advantage or disadvantage to either method with respect to pain provocation. However, this sample comprised people with a variety of different pain conditions. It is possible that a population with only hip or knee pain would have experienced a difference between the 2 tests, perhaps due to the increased frequency of turning in the conventional 6MWT. Moreover, pain was only assessed immediately after the walking tests, and both tests were completed 15 minutes apart; therefore, potential differences in pain experienced in the hours or days after the tests are beyond the scope of this study.

### Strengths and Limitations

This study compared Timed Walk against a conventional 6MWT on the same day. People with persistent pain can have variability in day-to-day pain [66,67], which may result in variations in walking speed. Conducting the tests 15 minutes apart removed this source of variability. However, the pain caused by the first 6MWT was clearly still a factor during the second 6MWT for some individuals. It is unclear whether extending the rest period to 30 minutes or an hour would have been sufficient for this pain to resolve completely. Moreover, only pain intensity was assessed and not the quality of the pain. Recording descriptors of the quality of pain (such as sharp, dull, throbbing, or aching) or assessing pain-related fear of movement might have provided deeper insights into why some participants with significant pain increases did not exhibit changes in walking distance but others did.

The study used the Timed Walk app on participant’s own smartphones. Similarly, this study used a wide variety of heterogenous walking tracks on grass or paved surfaces at a local park, with this heterogeneity precluding any analysis of the effect of walking track on the results. Although the observed agreement between methods may have been higher if a single smartphone and a set walking track were used consistently for all GPS-based walking tests, the results of this study have more practical relevance to people using their own smartphone in real-world environments. Similarly, as GPS is reported to have issues in measuring distances around sharp corners, a linear track for GPS may have been a more accurate measure of distance walked but would have been practically more difficult for participants to replicate at home and thus would be of less practical relevance.

This study calculated the 95% LoAs and their CIs under the assumption that the data were normally distributed. However, due to noticeable kurtosis (excess kurtosis of 2.2), the calculated 95% LoAs may be a slight overestimate. Although a nonparametric approach found that the 2.5% and 97.5% quantiles of the sample fell just within the nominated maximum allowable difference, it did not provide CIs to represent the uncertainty in the 95% LoAs. To obtain these CIs, the data could have been transformed to better approximate a normal distribution, or a bootstrap approach could have been used. However, regardless of the approach used, it is likely that the 95% LoAs or the associated CIs would be outside the maximum allowable difference.

The maximum allowable difference nominated in this study was selected in the absence of clearly established values for clinically relevant differences in the 6MWT in persistent pain conditions. A value of 100 m was selected as a compromise between the existing MCID estimates for the 6MWT in people with chronic pain of the back and lower limbs (60 m and 75 m, respectively) [19] and in those with fibromyalgia (which range from 156 to 167 m) [29]. The lack of knowledge about clinically acceptable differences for the 6MWT significantly limits the interpretation of the Bland-Altman analysis.

### Implications for Future Studies and Clinical Practice

Future studies are required to develop more accurate methods for remotely performing the 6MWT in populations with chronic disease including persistent pain. This may involve accelerometer-based methods, GPS-based methods, or a combination of both. Future updates to the Timed Walk app will require reassessment of concurrent validity. In addition, future studies could further elucidate the sources of variability between the GPS-based 6MWT and conventional 6MWT. This could entail performing a GPS-based 6MWT with the additional use of a trundle wheel as a third measurement of the 6MWD, simultaneous measurements derived from multiple smartphones from various manufacturers, and a systematic comparison of specific walking tracks. It may also be wise for future research studies to ensure that participants unfamiliar with the 6MWT are given a practice test to minimize the potential learning effect. Finally, more research is needed to establish a more precise estimate of the MCID in persistent pain to allow for future studies to better define the maximum allowable difference between 6MWT methods.

As both tests in this study were performed under supervision, future investigations should consider the effect of unsupervised use of the GPS-based 6MWT app. Tests that are self-administered may be even more variable if care is not taken to avoid sharp turns, tall buildings, uneven terrain, or inconsistencies in weather conditions. Future studies are also required to investigate whether the test-retest reliability of the GPS-based 6MWT, when used unsupervised in people with persistent pain, is sufficient for use as a tool for remote monitoring. It is feasible that the imprecision in the estimates from the GPS-based app may be less of an issue if testing is conducted more frequently, as the average of a series of tests may provide a more stable estimate of the participant’s functional status over time.

Although conventional and GPS-based 6MWT methods do not demonstrate sufficient agreement to be used interchangeably in people with persistent pain, the ability to perform the test remotely using the GPS-based app may still have benefits in a clinical context. The ability for patients to perform the GPS-based assessment at home without the need for a clinician’s presence may increase patient autonomy and reduce the burden of frequent clinic visits. In addition, for individuals who are unable to regularly attend in-clinic evaluations, obtaining an approximate measurement of functional capacity more frequently via a GPS-based 6MWT may still be clinically useful, even if the estimate is imprecise, as it provides some information, which is better than a complete lack of data. GPS-based measurements may also offer more ecologically relevant assessments of functional capacity than in-clinic testing, better reflecting a patient’s natural walking abilities and providing an opportunity for training walking capacity in the patient’s everyday environment. Overall, although the in-clinic standard 6MWT performed by experienced personnel remains as the gold standard, the GPS-based app may still be considered as a complementary tool.

### Conclusions

This study demonstrated that the concurrent validity between the GPS-based 6MWT using the Timed Walk app and the conventional 6MWT may not be sufficient for the 2 methods to be used interchangeably in people with persistent pain while still being able to detect clinically significant differences in the 6MWD. Despite limited validity, the GPS-based 6MWT may still have clinical application as a complementary tool to the conventional 6MWT performed in the clinic, especially for remote monitoring. In addition, the GPS-based 6MWT makes it possible to conduct more frequent assessments of functional capacity, which are self-measured without the presence of a clinician and conducted in a more ecologically relevant environment. Future studies are needed to improve the accuracy of the GPS-based 6MWT for remote monitoring.

## Appendix

Multimedia Appendix 1 Bland-Altman plots depicting the difference between GPS-based and conventional 6-minute walk distances against the average of the 2 measurements, with linear regression trend lines estimated for either the whole sample or for each recruitment group.

Multimedia Appendix 2 Scatter plots depicting the difference in walking distance between the 2 pairs of tests (GPS-based test vs conventional test and type of walk test conducted first vs type of walk test conducted second) against the difference in pain levels at the start of each walk test.

## Abbreviations

6MWD: 6-minute walk distance
6MWT: 6-minute walk test
ICC: intraclass correlation coefficient
LoA: limit of agreement
MCID: minimum clinically important difference
NRS: numerical rating scale

## Data Availability

The data sets generated during and analyzed in this study are not publicly available due to lack of ethical clearance to disclose data to third parties.
