# Supplementary material for: Assessing a GPS-Based 6-Minute Walk Test for People With Persistent Pain: Validation Study
Source: JMIR Form Res. 2024 Mar 18;8:e46820. doi: 10.2196/46820 (PMC10985605; doi:10.2196/46820)
Supplement: Multimedia Appendix 1 [file formative_v8i1e46820_app1.pdf]

## Multimedia Appendix 1

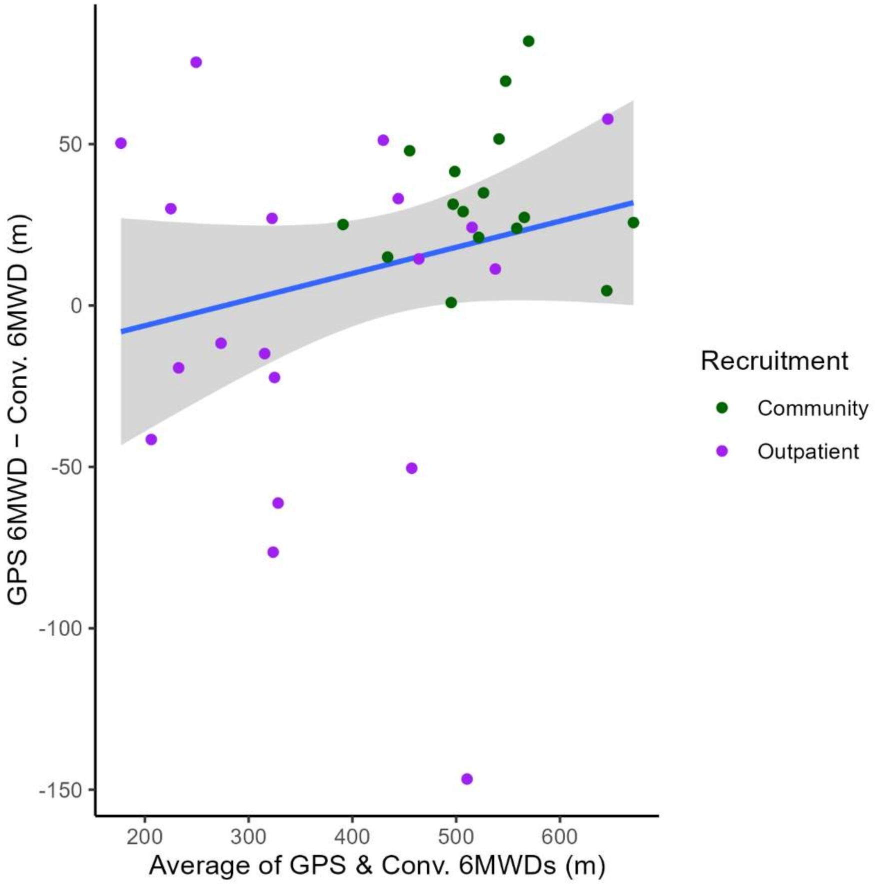


Bland-Altman plot depicting the difference between GPS-based and conventional (Conv.) 6MWD against the average of the two measurements, with community participants in dark green and outpatient participants in purple. The blue line represents the linear regression trendline of the mean difference between the two methods across the range of average distances, with shaded grey area representing the 95% confidence interval for the linear regression trend line. 6MWD: 6-minute walk distance; GPS: global positioning system

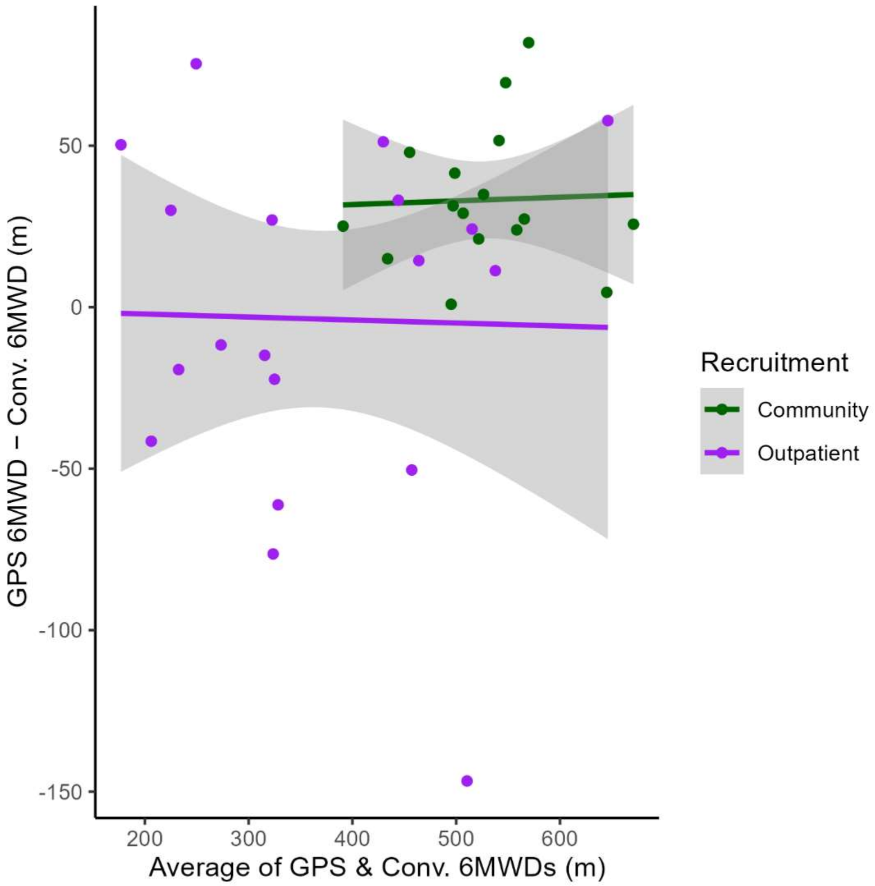


Bland-Altman plot depicting the difference between GPS-based and conventional (Conv.) 6MWD against the average of the two measurements, with community participants in dark green and outpatient participants in purple. The solid lines represent the linear regression trendline for each recruitment group, illustrating the relationship between the average 6MWD and the differences between the two 6MWDs. The shaded grey area represents the 95% confidence interval for the linear regression trend lines. 6MWD: 6-minute walk distance; GPS: global positioning system
