# Supplementary material for: Assessing a GPS-Based 6-Minute Walk Test for People With Persistent Pain: Validation Study
Source: JMIR Form Res. 2024 Mar 18;8:e46820. doi: 10.2196/46820 (PMC10985605; doi:10.2196/46820)
Supplement: Multimedia Appendix 2 [file formative_v8i1e46820_app2.pdf]

Multimedia Appendix 2

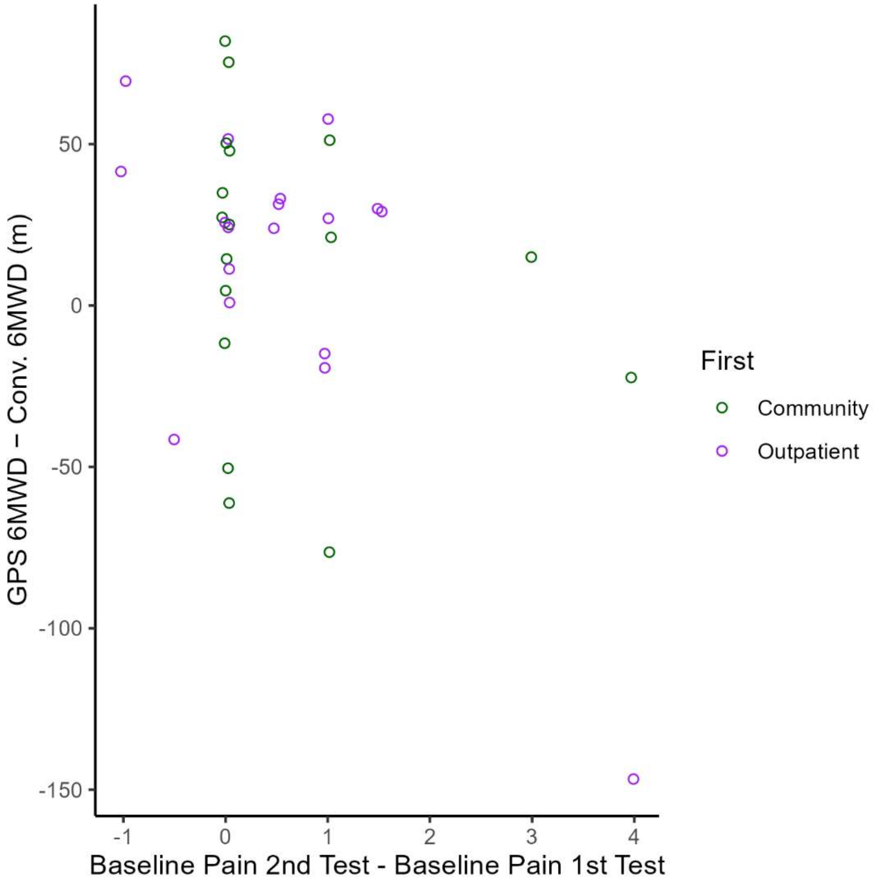

Scatter plot depicting the difference between GPS-based and conventional (Conv.) 6-minute walk distance (6MWD) against the difference between pain levels at the baseline (start) of each walk test. Community participants are represented as dark green circles and outpatient participants in purple. A slight horizontal jitter has been applied to aid in distinguishing overlapping data points.

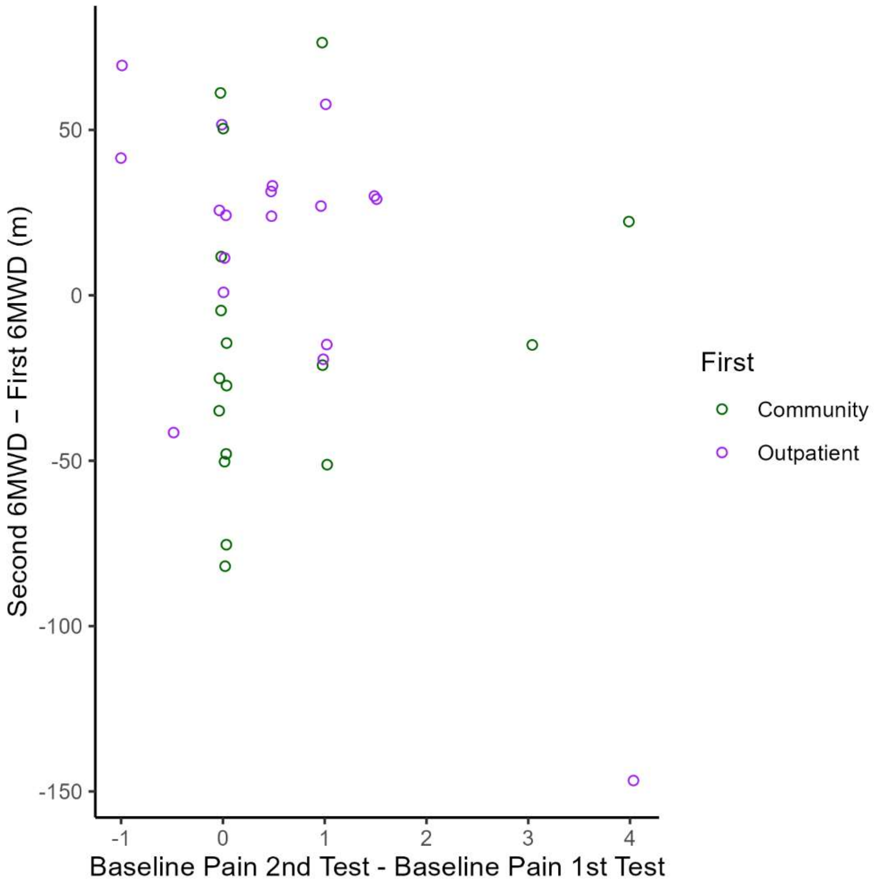

Scatter plot depicting the difference between 6MWD achieved on the first and second walk tests, against the difference between pain levels at the baseline (start) of each walk test. Community participants are represented as dark green circles and outpatient participants in purple. A slight horizontal jitter has been applied to aid in distinguishing overlapping data points.
